# Cross study transcriptomic investigation of Alzheimer’s brain tissue discoveries and limitations

**DOI:** 10.1038/s41598-025-01017-y

**Published:** 2025-05-08

**Authors:** Fernando Koiti Tsurukawa, Yixiang Mao, Cesar Sanchez-Villalobos, Nishtha Khanna, Chiquito J. Crasto, J. Josh Lawrence, Ranadip Pal

**Affiliations:** 1 Department of Electrical and Computer Engineering, Texas Tech University, Lubbock, TX 79409 USA; 2 Center for Biotechnology and Genomics, Texas Tech University, Lubbock, TX 79409 USA; 3 Department of Pharmacology and Neuroscience, Garrison Institute on Aging, Center of Excellence for Translational Neuroscience and Therapeutics, and Center of Excellence in Integrative Health, Texas Tech University Health Sciences Center, Lubbock, TX 79430 USA

**Keywords:** Alzheimer’s disease, Multivariate analysis, Machine learning, RNA sequencing, Transcriptomics, KCNIP1, Computational science, Neurodegenerative diseases, Learning algorithms

## Abstract

Developing effective treatments for Alzheimer’s disease (AD) likely requires a deep understanding of molecular mechanisms. Integration of transcriptomic datasets and developing innovative computational analyses may yield novel molecular targets with broad applicability. The motivation for this study was conceived from two main observations: (a) most transcriptomic analyses of AD data consider univariate differential expression analysis, and (b) insights are often not transferable across studies. We designed a machine learning-based framework that can elucidate interpretable multivariate relationships from multiple human AD studies to discover robust transcriptomic AD biomarkers transferable across multiple studies. Our analysis of three human hippocampus datasets revealed multiple robust synergistic associations from unrelated pathways along with inconsistencies of gene associations across different studies. Our study underscores the utility of developing AI-assisted next-gen metrics for integration, robustness, and generalization and also highlights the potential benefit of elucidating molecular mechanisms and pathways that are important in targeting a single population.

## Introduction

Alzheimer’s Disease (AD) is a complex neurodegenerative disorder characterized by amyloid plaques, neurofibrillary tangles, and progressive cognitive decline^1^. This disorder poses a true public health challenge due to its increasing prevalence among the growing aging population. Recently, RNA sequencing (RNA-Seq) has emerged as a dominant, powerful gene expression quantification technique. Transcriptomic analyses have provided critical revelations into biological mechanisms that underlie AD pathology. Typically, in AD, RNA transcripts responsible for immune response are upregulated, whereas pathways related to synaptic transmission and cell-to-cell signaling are downregulated^2,3^. The analysis of RNA-Seq data commonly uses tools such as edgeR^4^ and DESeq2^5^, which implement univariate statistical tests such as differential expression (DE) analysis. These univariate statistical tests rank gene relevance according to their significance (p-value) and fold change (FC). Transcriptomic studies typically apply differential expression analysis due to its scalability, robustness, and interpretability, which are crucial aspects for identifying new gene-based biomarkers^6^. However, beyond comparing DE analysis, emerging new studies have begun to demonstrate the importance of multivariate methods for exploring the AD transcriptomic landscape^7–12^.

Multivariate methods using typical machine learning (ML) approaches have been gaining ground in disease classification and have shown to perform well in AD^13^. For instance, Random Forest (RF) based methods can render classification accuracy of 91.6%^14^ and correlation-based feature selection followed by Support Vector Machines (SVM) can achieve 94% accuracy^15^ using transcriptomic data. Under specific scenarios with increased number of features, even scores in the 99% accuracy range have been reported^16^. However, an increasing number of features in a machine learning model can lead to over-fitting. Moreover, neural network-based classifiers can obtain accuracy scores above 90%^9^, although interpretation is limited.

In this paper, we designed and report the results of AD classification using simpler bivariate models, resulting in more interpretable and robust gene-based biomarkers. We considered classification using transcriptomic data to arrive at a set of novel, gene-based biomarkers that can potentially be used for targeted therapeutic development.

The hippocampus is known to be a critical region of the brain when it comes to AD^17^. In typical AD progression, this area is affected very early on, showing rapid atrophy rates^18^. The dysfunction spreads to surrounding tissues, eventually leading to generalized loss of neuronal activity and collapse of critical mechanisms in the brain. Most studies focus on specific regions of the brain, for instance Park et al. (2018) analyze gene expression profiles from the prefrontal cortex (PFC)^14^, while Zhang et al. (2013) worked with the cerebellum and the visual cortex^19^. The prefrontal cortex is also known to be dysregulated, particularly in the case of late-onset AD^3^.

There are limited studies in the literature regarding the integrated analysis of multiple AD datasets to elucidate robust biomarkers, particularly in the human hippocampus. Validating results from multiple studies is vital for capturing nuances in different populations and verifying promising gene-based biomarkers before constructing hypotheses and investigating their underlying biological mechanisms. Typically, validating results from different datasets involves checking the directionality of the fold change in DE analysis, while observing statistical requirements, such as false discovery rates. Multivariate non-parametric models lack concepts of directionality, fold change and statistical significance. Therefore it is imperative to be cautious when drawing conclusions from such models.

The current manuscript considers the design of a robust machine learning pipeline to analyze hippocampus transcriptomic datasets from three independent studies using two different measuring approaches: RNA-Seq and microarray. The insights gained from the analysis are described, along with caveats in interpretation and applicability. We also considered pathway analysis to explore the biological relevance of the results learned from the integrated analysis of the three datasets. Finally, the robustness of the inferred biomarkers were evaluated by simulating the experiments with permuted datasets to create the null distribution of feature importance scores.

This article is organized as follows. Section “Results” provides a description of the results of the analysis. Section “Discussion” provides a discussion of the results and the gathered insights. The methods used in the manuscript are provided in section “Methods”.

## Results

Our study utilizes three datasets, two of which employ RNA-Seq and one that employs microarray technology. The first is an RNA-Seq gene expression dataset provided by van Rooij et al. (2019)^20^ and shall be henceforth referred as VR. The second is also an RNA-Seq dataset, part of the Mayo Clinic Hippocampal Vulnerability Study^21^ and shall be referred as MAYO dataset. The third dataset is the only one that comes from microarray analysis and is obtained from Gene Expression Omnibus, identifier GSE29378^2^, and will be referred as GSE dataset. The three datasets were selected prioritizing the region of the brain where the tissue was extracted. The data collection and preprocessing techniques are documented in section “Datasets”. The demographic data, DE expression results and directionality of fold change are explained in the Supplementary Information, but are not included in the main text due to our study not relying on DE analysis.

### Data visualization

The plotted Uniform Manifold Approximation and Projections (UMAPs) in Supplementary Fig. S1 before and after the feature selection indicate better class separation between case and control samples after application of feature selection as compared to without feature selection. The same pattern is observed using t-distributed stochastic neighbor embedding (tSNE) and Principal Component Analysis (PCA), as shown in Supplementary Figs. S2 and S3, respectively.

### Bivariate analysis

**Fig. 1:**
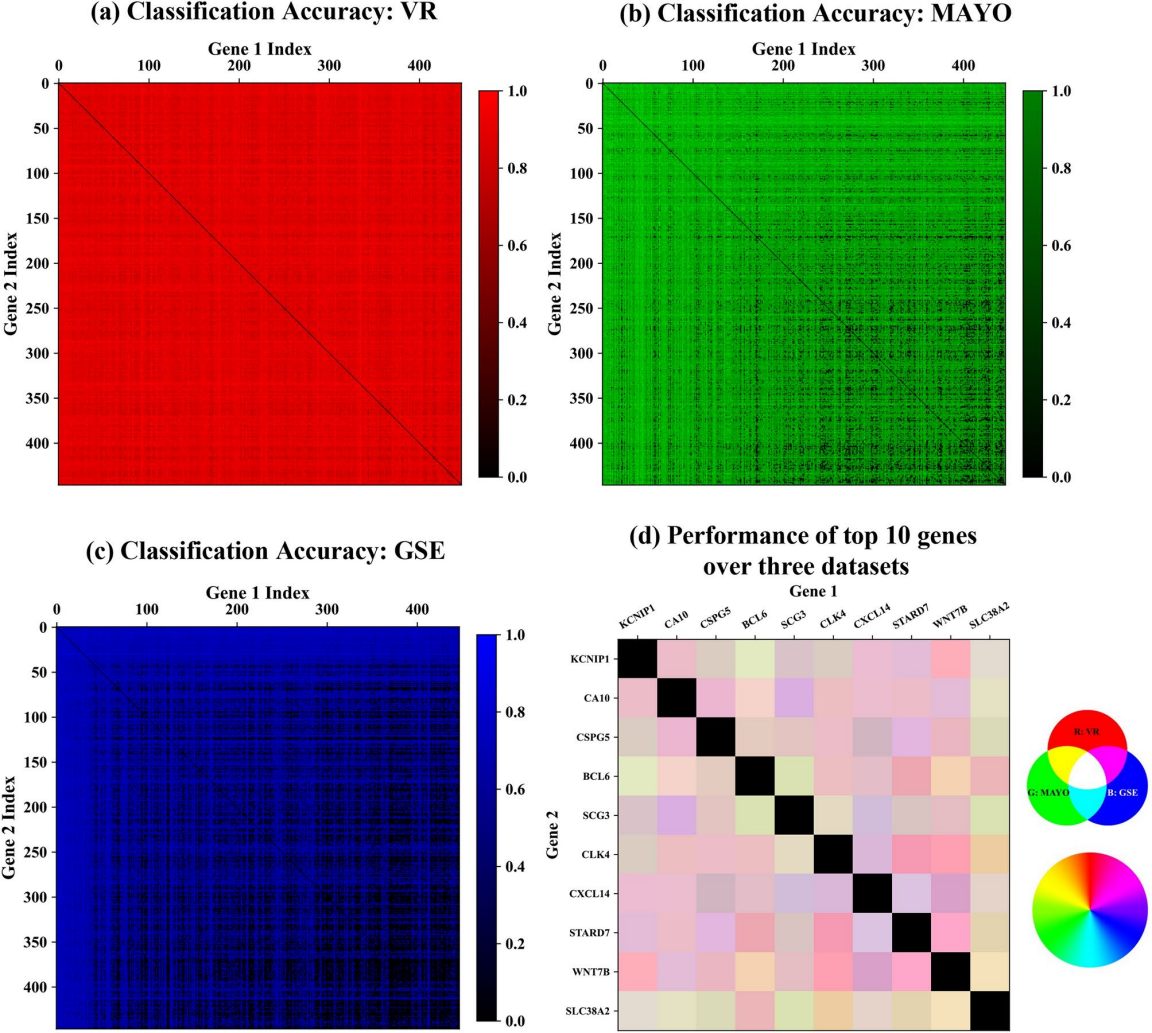
RGB-encoded visualization of bivariate gene pair accuracy scores. This figure presents a visual representation of accuracy scores from all possible combinations of the top 10 ranked genes in our bivariate ranking, using an RGB-encoded pixel format. In panels (**a**)–(**c**), the accuracy scores for each hippocampal dataset—VR, MAYO, and GSE—are depicted in distinct color schemes: red for VR, green for MAYO, and blue for GSE. Panel (**d**) merges these representations additively. A pixel with a white color indicates a perfect accuracy score across all three datasets, signifying robust gene pair performance. The diagonal encodes the combination of a gene with itself and is not relevant for this analysis.

The results of applying the bivariate analysis using SVMs are presented in Fig. 1, which displays a pixelated visualization of the gene pairs performances across the three different datasets in a color-coded fashion. This visual representation serves to display the superiority of the top 10 ranked genes from our bivariate ranking approach. Each pixel’s intensity represents a score obtained from training an SVM. In the monochromatic panels (a, b, c) the pixel intensity corresponds to the cross-validation accuracy of a gene pair in a single dataset. Panels (a, b, c) illustrate that there are numerous pairs with high scores in VR—primarily red pixels in (a)—as compared to MAYO and GSE (large number of black pixels denoting low accuracy in (b) and (c). Note that there are substantially more black pixels in GSE as compared to MAYO). The polychromatic panel (d) displays the performance across all three datasets by combining the monochromatic panels additively, shown for only the top 10 ranked genes. Most gene combinations present pink colored pixels, which encode a dominance of high scores in VR (red). The gene *SLC38A2* displays more beige colored pixels, which encode a more balanced combination of scores. By assessing the accuracy of gene pairs in multiple datasets, we can identify biomarkers that are not only highly accurate but also generalizable across different populations and conditions.

**Fig. 2:**
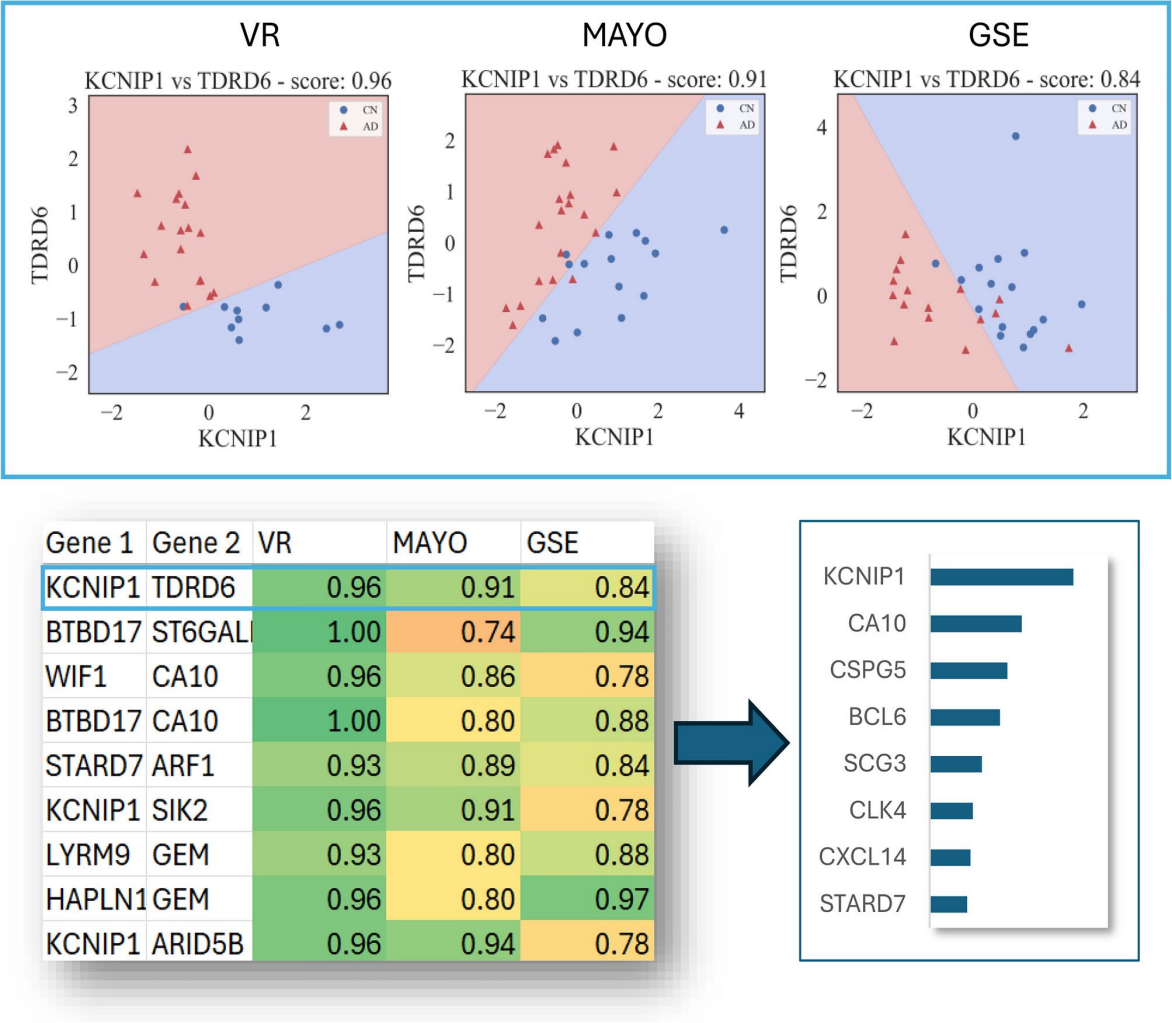
Considered approach for ranking gene importance in Alzheimer’s disease from multiple datasets. This figure illustrates the methodologies employed for ranking gene importance using bivariate and multivariate approaches across hippocampal transcriptomic datasets. Bivariate ranking methodology is used to exhaustively search for top performing gene pairs. All pairs from the top 500 genes from Relief feature selection (124,750) are evaluated across three hippocampal transcriptomic datasets. The accuracy scores above the 0.70 threshold are added up, producing a bivariate ranking.

Among the three datasets, we observe that the average performance of a gene pair selected in one dataset tends to be lower when tested in other datasets, as shown in Supplementary Fig. S4. The average CV score for gene pairs selected from the VR dataset is 0.89, while the average performance of these same gene pairs is 0.63 in the MAYO dataset and 0.57 in the GSE dataset, significantly lower with $p < 0.001$ measured by a Welch’s t-test. This disparity reveals potential dataset-specific variations in gene expression patterns. The significantly higher CV scores in the VR dataset compared to MAYO and GSE highlight the importance of considering multiple datasets to capture a more comprehensive view of the disease. Analysis performed using only a single transcriptomic dataset might fail to encompass the entire panorama of Alzheimer’s Disease due to limited sample sizes and differences in population characteristics. For example, the VR dataset includes reads from the Netherlands Brain Bank, while the MAYO dataset contains reads from the Mayo Clinic Florida Brain Bank. To address this, we evaluated the performance of gene pairs across all three datasets, providing a more robust and generalizable assessment of gene pair relevance, following the approach from Fig. 2.

To demonstrate the advantages of bivariate analysis over univariate differential expression analysis, we present the fold changes of the top genes identified by the bivariate methodology in Supplementary Table S1. This table lists the aggregated scores from the top-ranked genes based on our bivariate ranking methodology, along with their respective fold changes in each of the three hippocampal studies. A key observation is that many of the top bivariate genes are not unanimously differentially expressed in all three datasets and would have been potentially overlooked if differential expression analysis alone was used. For instance, *KCNIP1*, the top-ranked gene from our bivariate analysis, demonstrates high aggregated scores across the datasets, showcasing its relevance despite not being consistently differentially expressed in univariate analysis. This highlights the innate difference between our bivariate methodology, which captures gene interactions and their combined effects, and the commonly accepted univariate approach of differential expression analysis, which evaluates each gene in isolation.

The differences between DE analysis and the bivariate analysis is further shown in the Supplementary Information. The volcano plot displayed in Supplementary Fig. S5 shows the statistical significance of the differential expression analysis of the two RNA-Seq gene expression profiles. The volcano plot in Supplementary Fig. S6 displays the statistical significance of the differential expression analysis in the microarray study. The location of the top 10 ranked genes in the volcano plots reveals that most of them are not significantly differentially expressed in MAYO and GSE.

### Potential biomarkers

**Fig. 3:**
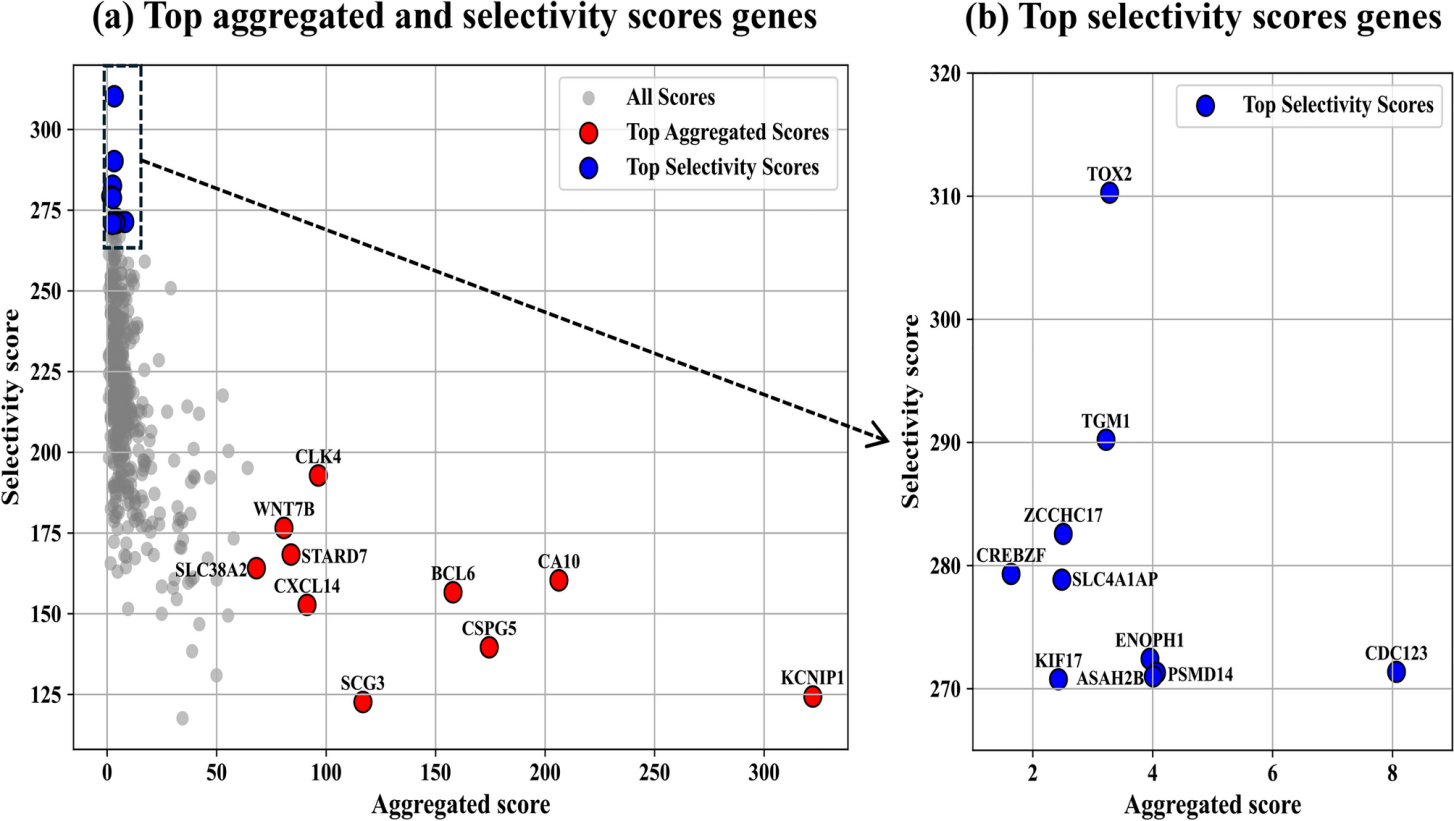
Scatter plot of aggregated scores vs. selectivity scores for gene pairs. This scatter plot presents the relationship between aggregated scores and selectivity scores for gene pairs in our bivariate analysis. The aggregated score, plotted on the x-axis, reflects the robustness of gene pair performance across multiple hippocampal datasets, with higher scores indicating consistent accuracy across VR, MAYO, and GSE datasets. The selectivity score, shown on the y-axis, measures the discrepancy in performance across these datasets, with higher selectivity scores indicating greater variability.

Among the top scoring genes, we highlight *KCNIP1* ($\text {CV\_sum}=322.22$), *CA10* ($\text {CV\_sum}=206.29$), *CSPG5* ($\text {CV\_sum}=174.48$), *BCL6* ($\text {CV\_sum}=157.95$) and *SCG3* ($\text {CV\_sum}=116.80$). The bivariate gene ranking remains the same when perturbing the 0.70 threshold. We also observed that *SLC7A2* ($\text {CV\_sum}=127.95$) and *CMTM4* ($\text {CV\_sum}=94.00$) were only present when selecting the top 500 genes from Relief in MAYO. The top genes selected through aggregated and selectivity scores across three datasets are shown in Fig. 3.

In VR, most of the genes in our bivariate ranking perform well by themselves, obtaining 90% accuracy scores or higher without being paired to any other gene. As an exception, the genes *RLBP1*, BRINP3, *C17orf58*, *TRIB1* and *SHISA4* have poor accuracy scores individually, but perform considerably better when paired with other genes.

In MAYO, the top genes from our ranking are not able to split the classes by themselves and gain considerable accuracy when combined in pairs. Particularly, *SCG3*, *CLK4*, *STARD7* and *WNT7B* obtain accuracy scores below 0.70 if we used a univariate approach. The same pattern can be observed in GSE with the following genes: *BCL6*, *SCG3*, *CXCL14* and *WNT7B*. These genes are not differentially expressed in some studies like MAYO and GSE, but show more consistent performance when paired with other genes.

Supplementary Fig. S7 shows an example of a robust gene pair of *KCNIP1* and *SLC38A2* across three datasets. The performance of this pair is higher in VR and MAYO datasets while being slightly lower in GSE. Furthermore, the slopes of the decision boundary shows the importance of both the genes for classification in the VR and MAYO whereas limited gain is achieved from adding *SLC38A2* to *KCNIP1* in GSE dataset. This variation could be due to the different sample distributions in the dataset or due to the technology used (microarray for GSE as opposed to RNA-Seq for VR and Mayo).

Supplementary Fig. S8 shows an example of a more dataset-dependent gene pair of *RBP1* and *WNT7B*. The gene pair provides perfect separation in the VR dataset but poor separation in the MAYO dataset and relatively lower separation in GSE.

### Robustness analysis

We observed a high level of agreement between the list of genes selected before and after adding the noise (Kendall Rank Correlation coefficient of $\tau = 0.94$). Similar agreement in rankings were observed in our bivariate analysis pipeline ($\tau = 0.93$).

The rank correlation coefficient $\tau$ for the rankings using thresholds 0.7 and 0.65 ($\tau =0.89$) and between the rankings using thresholds 0.70 and 0.75 ($\tau =0.82$). These findings suggest that our rankings demonstrate robustness across different threshold choices, indicating consistent outcomes regardless of the specific threshold used.

**Fig. 4:**
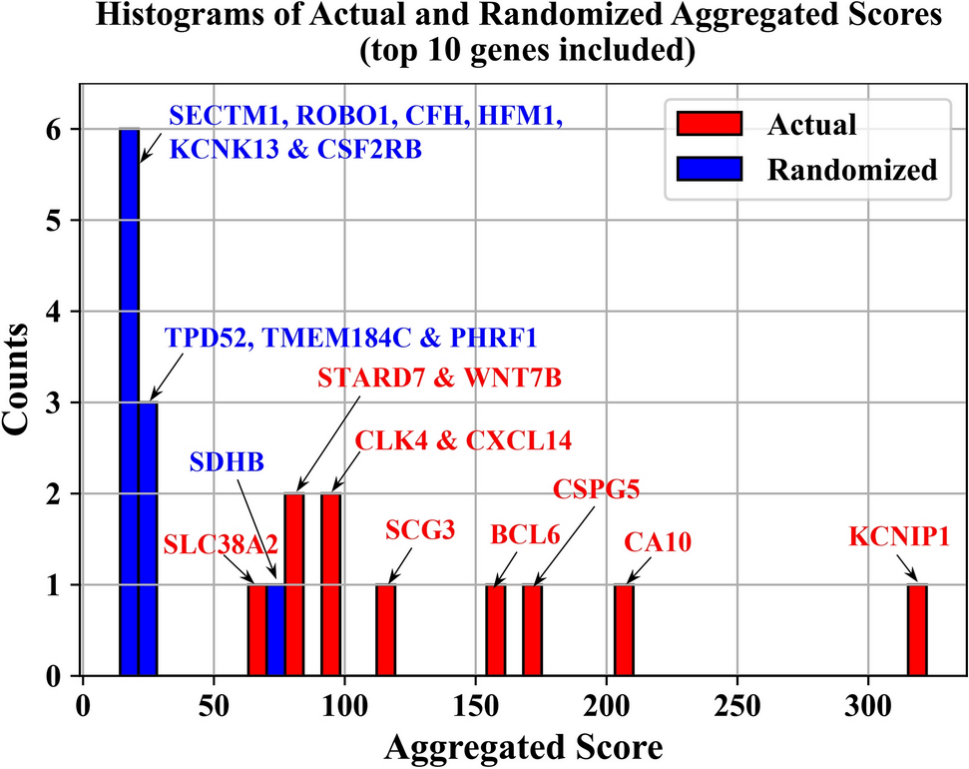
Comparison of actual and randomized aggregated scores. This figure presents histograms of the top 10 actual (red) and randomized (blue) aggregated scores. Randomized scores represent a quantified version of the null hypothesis, obtained by permuting the actual labels 200 times and repeating the bivariate score computation. These randomized scores help assess the statistical significance of the original ranking. By comparing the performance of the genes identified in the original analysis to these randomized scores, we can evaluate the likelihood that the original ranking occurred by chance. The top 10 randomized aggregated scores were selected from a distribution of 100,000 randomized scores. The histograms show that the genes from the original analysis, particularly KCNIP1, which is the top-ranked gene, perform significantly better than top-ranked genes in the randomized scenario. This indicates that the original ranking is statistically significant and that KCNIP1’s high rank is not due to random chance.

The majority of the randomized aggregated scores are close to zero, as shown in Supplementary Fig. S9. For visualization, we selected the top 10 randomized aggregated scores from permutation testing and compared them with the actual aggregated scores. As shown in Fig. 4, even the smallest actual aggregated score (for gene *SLC38A2* at 68.16) is almost on pair with the largest randomized aggregated score (for gene *SDHB* at 76.62). All other actual aggregated scores exceed the randomized scores. Given the total of 100,000 randomized aggregated scores, these results indicate the possibility of observing the actual aggregated scores by chance is very low ($p=1\times 10^{-5}$ for gene *SLC38A2* and $p<1\times 10^{-5}$ for the other genes).

## Discussion

Transcriptomic data may have multiple interpretations, depending on the tissue being analyzed and the technique applied to extract gene expression. Next Generation Sequencing (NGS) data has the advantage of being more general than microarray data, as microarray experiments require a priori strands of cDNA for the analysis. In this multi-study, two datasets—VR and MAYO—were extracted using modern NGS techniques, while the last dataset—GSE—applies microarray. Microarrays rely on pre-designed probes, limiting their ability to detect less abundant genes. Furthermore, microarray technology has to deal with a limited range and suffers from background noise. In contrast, RNA-Seq offers an unbiased approach by sequencing all RNA molecules present in a sample.

Differential expression results for each of the three hippocampal transcriptomic datasets vary substantially in terms of fold change; however, the directionality of the most significant genes remains the same. The assumptions of differential expression analysis may not align with the objectives of machine learning, where predictive performance relies on capturing diverse patterns beyond fold changes in gene expression. One novel aspect of our study is that univariate feature selection approaches, such as Relief-F, manage to extract genes that are not significantly differentially expressed but seem to have undiscovered associations with AD. Focusing on gene pairs, rather than individual genes, the application of Relief-F produces a set of genes of interest that further diverges from conventional DE analysis and incorporates potential synergistic relationships among genes, providing potential therapeutic targets for future research. The contrast between Relief-F and conventional DE analysis is further displayed in Supplementary Table S2, which shows that among the 500 selected genes from applying Relief-F in VR transcriptomes, only 22% of those are considered significantly differentially expressed.

Another aspect of AD classification tasks lie on the kind of data being used for model training. Note that MRI image based classification approaches have shown to achieve higher accuracy as compared to transcriptomic analysis^22–24^. This is likely due to the fact that AD affects brain memory and has a strong impact on brain volume. Using gene expression data for AD classification tasks presents both opportunities and limitations. AD is a complex disorder in which the relationships between cause and effect are often intertwined. Dysregulation of the transcriptome struggles to tell apart the underlying molecular mechanisms from the compensatory responses of the body. AD is typically characterized by extracellular deposition of amyloid $\beta$ (A$\beta$) and intracellular neurofibrillary tangles, combined with a progressive decline of neuronal function^25^. This triggers a strong response from the brain, indicated by the upregulation of genes related to immune response activation.

The identification of potential gene-based biomarkers for Alzheimer’s disease through our robust machine learning pipeline reveals the potential for uncovering undocumented insights into AD pathogenesis. We further evaluated the robustness of our approach through multiple approaches and arrived at some potential new biomarkers for AD. The discovery of these novel associations highlights the importance of continuing research efforts to explore the complex interactions between genetic modifications and neurodegenerative diseases. Further investigation and validation of these novel associations are essential for advancing our understanding of AD and expanding the scope of transcriptomic research in the context of neurodegenerative disorders. We developed approaches to determine applicability across datasets and observed highly differentiating behavior using multiple gene pairs in one dataset which were not replicated in the other datasets. The limited applicability of some gene pairs may offer insights into molecular mechanisms and targets that may be applicable to only one population, which could be a new frontier in precision medicine.

Identifying prognostic gene pairs using transcriptomic data presents several challenges and caveats that need careful consideration. The inherent complexity and variability of transcriptomic data can lead to both promising discoveries, but also potential misinterpretations.

One interesting result is the observation of synergistic associations between gene pairs that don’t belong to the same biological pathway. For instance, the genes *KCNIP1* and *SLC38A2* have shown combined association with AD, despite these genes not being traditionally linked through the same biological processes. This might suggest the existence of a novel pathway or an intricate network of interactions that implicates both genes in the progression of AD. Such findings can open new avenues for research, hinting at undiscovered mechanisms that contribute to the disease. However, it requires further validation and functional studies to confirm these interactions and elucidate their roles in AD pathology.

*KCNIP1* belongs to the family of voltage-gated potassium channel-interacting proteins (*KCNIPs*) that regulate the A-type currents and neuronal excitability in response to changes in intracellular calcium^26^. *KCNIP1* was nominated as an AD gene in 2018 by Dr. Rima Kaddurah-Daouk (Duke AMP-AD team). *KCNIP1* interacts directly with the N-terminal domain of Kv4 channels, modulating its cell surface expression and function^27^. Distinct from its effects on Kv4.2-mediated channels, *KCNIP1* accelerates the inactivation of Kv4.1 channels and causes a depolarizing shift in the voltage dependence of activation, highlighting the complex and subtype-specific nature of *KCNIP1*’s regulatory effects on Kv4 channels^27^. In the absence of *KCNIP1* expression, Kv4 channels aggregate and misfold, and are consequently retained in the endoplasmic reticulum (ER) for degradation^28^. Kv4.1 downregulation has been shown in the dentate gyrus of AD mouse model, accompanied by granule cell hyperexcitability and impaired cognitive performance^29^. These findings suggest a potential role for *KCNIP1* in human AD^2,30^. *KCNIP1* knockout mice also exhibited increased susceptibility to seizures, as demonstrated by enhanced anxiety-like behavior and altered GABAergic neurotransmission^31^. Beyond its role in channel regulation, *KCNIP1* has emerged as a calcium-dependent transcriptional repressor. This function involved binding to a specific DNA sequence known as the Downstream Regulatory Element (DRE) sites, which are typically located in the promoter region of the target gene^32^.

*CA10*, also known as carbonic anhydrase X, is a metalloenzyme containing zinc necessary for ion transport across cellular membranes^33^. *CA10* can bind to all neurexin isoforms with high affinity^34^. Neurexins are a family of cell adhesion proteins in the neuronal presynaptic terminal. These proteins have been shown to play a role in synaptogenesis by interacting with postsynaptic ligands, such as neuroligins or leucine-rich-repeats transmembrane proteins (LRRTMs)^35^. A$\beta$ oligomers disrupt neurexin function, potentially contributing to synaptic dysfunction and cognitive decline in AD^36,37^. *CA10* has no well-established association with AD.

DeWitt et al. (1993)^38^, demonstrated that *CSPG5* is present in both senile plaques and neurofibrillary tangles, the hallmark pathological feature of AD. The researchers used immunohistochemical techniques to identify *CSPG5* in brain tissue samples from AD patients^38^. Genome-wide comparison of gene expression in CA1 and CA3 regions of the hippocampus revealed that *CSPG5* transcript is downregulated in advanced AD^2^. A recent experimental study conducted on middle-aged mice focused on the effects of memantine, a drug used in AD treatment and suggested that memantine regulates *CSPG5* biosynthesis and degradation in the hippocampus^39^. This regulation was associated with increased densities of newborn granule cells and improved mice’s short- and long-term memory performance^39^. Importantly, when *CSPG5* was pharmacologically depleted, the cognitive benefits of memantine were impaired, suggesting that *CSPG5* is essential for the drug’s effects^39^.

*BCL6* (B-cell lymphoma 6) is a transcriptional repressor that plays a crucial role in the pathogenesis of diffuse large B-cell lymphoma (DLBCL) and other B-cell malignancies^40^ As a master regulator of germinal center (GC) B-cell development, *BCL6* facilitates the physiological genomic instability required for antibody affinity maturation^40^. Recent findings demonstrated that *BCL6* may protect against amyloid-$\beta$-induced neuronal damage AD^41^. Their study found that overexpression of *BCL6* in SH-SY5Y cells attenuated A$\beta$-42-induced increases in p-Tau levels and improved cell viability, suggesting that NCL6 can be established as a potential target for mitigating A$\beta$-related pathology in AD^41^. Researchers investigated the expression pattern of *BCL6* in normal human brains and AD^42^. They found that *BCL6* is expressed in isolated cortical neurons, cerebellar granule cells, scattered glial cells, and some cells of ependyma and choroid plexus^42^. However, *BCL6* expression was absent in neurofibrillary tangles and the nuclei of cells associated with amyloid plaques in AD brains^42^.

Secretogranin III (*SCG3*) plays a role in producing and moving dense-core secretory vesicles and is widely expressed in neuroendocrine tissues and the central nervous system. In Parkinson’s Disease (PD) research, *SCG3* is upregulated in astrocytes exposed to Parkinsonian toxins such as MPTP (1-methyl-1.2.3.6.-tetrahydropyridine), a major synthetic neurotoxin, suggesting its involvement in astrocyte activation and neuroinflammatory process^43^. In AD, dopamine levels and receptors are significantly reduced, particularly in the striatum and hippocampus^44^. The decrease in *SCG3*-Positive secretory granules observed in dopamine neurons in PD models suggests a potentially similar mechanism in AD, where impaired vesicle trafficking could contribute to reduced dopamine signaling.

*CLK4* belongs to the family of cdc2-like kinases (*CLKs*) containing four isoforms namely *CLK1*, *CLK2*, *CLK3* and *CLK4*. *CLK1* has been considered as a potential target for Alzheimer’s disease drug development^45^. The dysregulation of the processes regulated by *CLKs* has been linked to various diseases, including neurodegenerative diseases, Duchenne muscular dystrophy, inflammatory diseases, viral replication, and cancer^46^.

*CXCL14* (C-X-C motif chemokine ligand 14) is a chemokine involved in immune system regulation and inflammation, and emerging research suggests it may play a role in Alzheimer’s disease. For instance, *CXCL14* may influence microglial behavior^47^ and indirectly affecting AD progression^48^. Furthermore, prior research points to the the expression of *CXCL14* by single-bouquet cells in layer I (LI) of the somatosensory cortex during development of mice and loss of *CXCL14* can cause increased intrinsic excitability and neuronal complexity^49^.

*STARD7* is a lipid transport protein that facilitates the transfer of phosphatidylcholine to mitochondria. Impaired mitochondrial function has been considered as a contributing factor to neurodegenerative diseases^50^. Specifically, mutations in the *STARD7* gene have been linked to mitochondrial dysfunction and neurodegenerative diseases^51,52^.

*WNT7b* is a member of the *WNT* gene family, which consists of structurally related genes that encode secreted signaling proteins. A substantial amount of prior research links the Wnt signaling pathway to synaptic regulation and cognitive functions, indicating that disruptions in this pathway may contribute to cognitive decline and linked to neurodegenerative diseases such as Alzheimer’s disease. Notably, changes in the expression of critical Wnt pathway components have been observed in the brains of both Alzheimer’s disease (AD) patients and animal models, reinforcing the idea that the pathway is dysregulated in AD^53^.

*SLC38A2*, also known as *SNAT2*, functions as a sodium-dependent amino acid transporter that mediates the neuronal efflux of neural $\alpha$-amino acids across the blood-brain barrier and their uptake into neurons^54^. *SLC38A2* was identified as one of the genes associated with selective hippocampal vulnerability in AD^21^. Notably, this upregulation was not confined to the hippocampus alone but was observed across multiple brain regions examined in the study^21^. Under hyperosmotic stress, *SLC38A2* expression is significantly upregulated in an NF-$\kappa$B-dependent manner, and its overexpression attenuated cell death in medullary collecting duct cells^21^. Various AD-related factors, including amyloid-$\beta$ peptides and oxidative stress, also trigger NF-$\kappa$B activation^55^, suggesting its potential influence on *SLC38A2* expression^56^. Correlation analysis and received operating characteristic (ROC) curves have verified *SLC38A2* as a possible key target associated with AD immunity^57^. *SLC38A2* is also observed to create a steep concentration gradient for amino acids, particularly glutamine^58^. As a glutamine transporter, *SLC38A2* serves an important role in maintaining amino acid homeostasis in the brain^59^. Glutamine is essential for neurotransmitter synthesis, particularly for the production of glutamate and GABA, which are crucial for cognitive function^60^. Dysfunction in *SLC38A2* expression could potentially disrupt this balance, leading to alterations in neurotransmitter levels and signaling pathways that are critical for normal brain function^59^. The observation of an association between *KCNIP1* and *SLC38A2* may strengthen an association between hyperexcitability (*KCNIP1*) and neuroinflammation (*SLC38A2*).

A caveat that should be noted is the inconsistency of gene associations across different datasets. For example, while the gene pair *RBP1* and *WNT7B* may exhibit distinct distribution patterns and a strong association with AD in VR (Supplementary Fig. S8), these findings do not replicate in MAYO, even though both datasets are derived from the same brain region. This variability can arise from several factors, including differences in sample preparation and patient demographics. Such discrepancies highlight the importance of replicating findings in multiple, independent datasets to ensure the robustness and generalization of the results.

While the identification of prognostic gene pairs using transcriptomic data holds great potential for understanding the complexities of Alzheimer’s Disease, there is still much room for improvement. Synergistic associations between genes from unrelated pathways could point to novel, uncharacterized pathways involved in AD, calling for deeper investigation. Conversely, the inconsistency of results across different datasets highlights the need for rigorous validation and replication across studies.

## Methods

### Datasets

This study integrates transcriptomic data from three independent datasets: VR, MAYO, and GSE, each of which was processed using distinct methodologies. The demographic data of each dataset is in Supplementary Table S3.

The VR dataset consists of RNA-Seq data obtained from hippocampal samples of 20 Alzheimer’s disease (AD) cases and 10 cognitively normal controls. The RNA sequencing was conducted using an Illumina HiSeq2000 platform with a paired-end read length of 2 $\times$ 50 base pairs (bp). Data processing involved trimming, alignment, and transcript quantification against the GENCODE reference genome (version date; 2013-12-05). The original authors identified and removed two samples in the AD group deemed as outliers through Principal Component Analysis. We’re left with 10 samples in the control group and 18 samples in the AD group.

The MAYO dataset contains bulk RNA-Seq hippocampus data, extracted from a *postmortem* cohort selected by a single neuropathologist^21^. Specifics on hippocampus tissue section are not included in the metadata. The MAYO hippocampus data is available through the AD Knowledge Portal, identified as *syn32141161*. The raw sequence files can be obtained through agreement to the project’s data-specific terms of use. The MAYO dataset includes RNA-Seq profiles from 55 hippocampal samples, representing different AD subtypes and control cases. The 55 samples are distributed as follows: 15 cognitively normal controls, 10 hippocampal sparing AD, 10 limbic predominant AD and 20 considered typical AD cases. RNA was extracted, followed by ribosomal RNA depletion and library preparation. Sequencing was performed on the Illumina HiSeq2500 platform, generating paired-end reads of 2 $\times$ 101 bp. Unlike the VR dataset, which we analyzed using preprocessed data, the raw reads from the MAYO study were processed *de novo* in this study using the DNASTAR software. Reads were mapped to the hg38 human genome assembly, and expression values were calculated in terms of Reads per Kilobase per Million (RPKM). Differential expression analysis applied a fold change threshold $\vert \log _2 FC \vert \ge 1$ and a significance threshold of $P < 0.05$, with subsequent false discovery rate (FDR) correction. The patients with extreme phenotype diagnosis were disregarded for this study and only the patients with typical AD diagnosis were accounted for our analysis, resulting in a cohort of 20 typical AD cases with 15 controls.

The third hippocampal transcriptomic dataset, which we will refer to as GSE, comes from a microarray transcriptomic study by Miller et al. (2013)^2^, available in NCBI’s Gene Expression Omnibus, accessible through Gene Expression Omnibus (GEO) Series GSE29378^2,61^. This study encompasses gene expression in advanced stage AD versus nondemented control patients in annotated regions CA1 and CA3. According to Miller et al. (2013), the CA1 region showed more significant gene differential expression. The GSE dataset is the only study that presents spatial annotation regarding the specific section of the hippocampus where the tissue was extracted. The GSE dataset (GSE29378) comprises microarray-based gene expression data obtained from hippocampal subfields. For this study, we focused exclusively on the CA1 region, as the original publication reported stronger differential gene expression in this subfield. Gene expression was profiled using the Illumina HumanHT-12 v3 Expression BeadChip, which covers over 25,000 genes. After quality control and outlier removal, 63 samples (31 AD and 32 control) remained in the analysis. Data preprocessing involved probe filtering based on expression levels, annotation status and redundancy, ultimately resulting in 17,128 unique gene expression profiles.

**Fig. 5:**
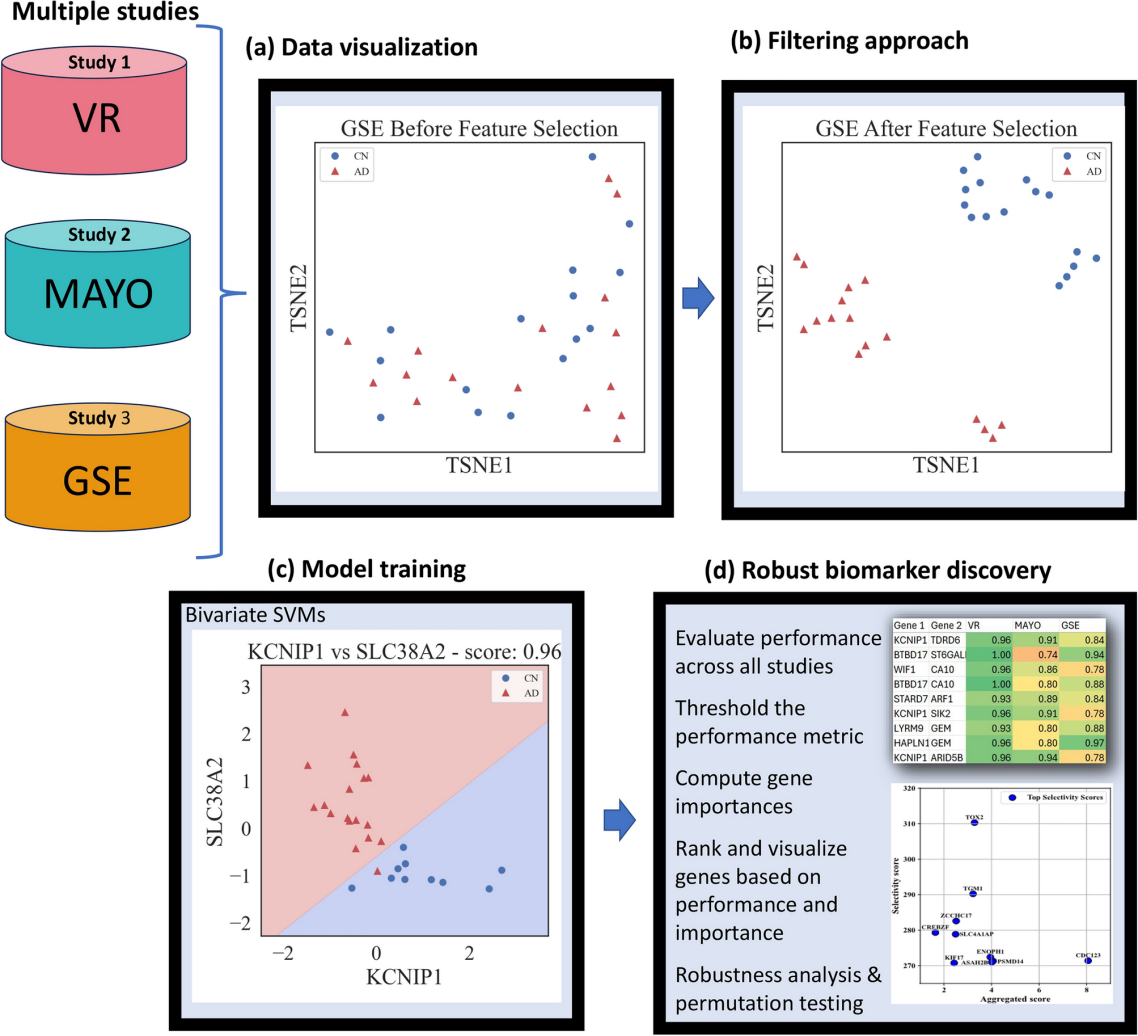
Workflow for biomarker discovery and pathway analysis across multiple studies. The process begins with (**a**) visualization of data from multiple studies (Study 1—VR, Study 2—MAYO, and Study 3—GSE). It proceeds with (**b**) the application of filtering approaches and (**c**) the training of predictive models using techniques such as SVM (**d**) Model performance is evaluated across all studies, with thresholds applied to performance metrics. Gene importance is computed, followed by the ranking and visualization of genes based on their performance and importance. The workflow includes robustness analysis and permutation testing to ensure the reliability of results.

The analysis workflow considered in this paper is shown diagrammatically in Fig. 5.

### UMAPs

To illustrate the importance of feature selection, we considered the representation of the entire multidimensional dataset in a lower dimensional manifold using Uniform Manifold Approximation and Projection (UMAP) and plotted the entire transcriptome data mapped to a lower manifold in Supplementary Fig. S1a–c before any feature selection. We then selected the top 500 ranked genes using filter feature selection approach Relief-F with its hyperparameter *k* set to 3^62,63^ and projected the multidimensional data once again in Supplementary Fig. S1d–f.

### Bivariate ranking using SVMs

For classification tasks, we utilized Support Vector Machines that are used to find the optimal hyperplane that separates different classes and minimizes classification error. We considered the exhaustive evaluation of all gene pairs from the 500 genes selected by the filter feature selection approach Relief-F. In order to evaluate the 124,750 gene pairs, we used SVM as a wrapper method to systematically assess each pair’s predictive power. We used linear SVMs in this scenario due to their robustness against overfitting and high interpretability. From the top 500 Relief-F genes from VR dataset, we exhaustively looked for the best gene pair in the reduced feature space by training linear SVMs ($C=100$) and computing its corresponding cross-validation score ($CV=5$) in each of our three hippocampal datasets. Keeping the gene pairs with CV scores above $70\%$, we’re left with 3428 gene pairs. At last, the scores for each gene were added up to rank the most relevant genes in bivariate classification of AD. The same process was repeated for the top 500 genes from MAYO and GSE. The aggregated score is the sum of the cross-validation scores from training SVMs across all three datasets. To ensure robustness, a threshold of 0.70 CV score was applied to exclude low-performing pairs before summing the scores.

### Feature selection

Differential expression analysis considers the importance of individual genes for categorizing the various groups and might miss out on the multivariate relationships that can differentiate the different classes. However, using all genes to categorize the different classes is also not an optimal solution. In fact, the sheer number of features compared to the small number of samples in transcriptomics poses a well-known machine learning problem called *curse of dimensionality*, where the performance increases with the number of features until it achieves a peak and then starts diminishing with further increase in dimensionality^64^. Consequently, machine learning approaches that are trained using all genes are often prone to overfitting and lack generalization power. Thus, we considered feature selection approaches to narrow down the genes being used for classification. We used Relief^65^ for initially reducing the number of genes. We also considered LASSO as a feature selector, are included in the Supplementary Information.

### Robustness analysis

We explored the robustness of our methodology in three different ways: Noise injection, where additive noise is added to the data and the variation in the gene ranking observed; Robustness to threshold selection and Permutation test, where permuted data labels are used to analyze the statistical significance of the gene ranking scores.

#### Noise injection

We investigated the robustness of our feature selection methodology, Relief-F, by injecting Gaussian additive noise with a standard deviation set to 0.05 times each gene expression’s standard deviation.

#### Threshold selection

To evaluate the robustness across different threshold choices, we compared three different choices of threshold by computing the Kendall Rank Correlation $\tau$ for the rankings using different thresholds. We evaluated the thresholds 0.65, 0.70 and 0.75 for bivariate ranking of the 500 selected genes from VR and evaluated the correlation between each ranking, taking into account its position in the rank as well as its score.

#### Permutation test

We performed permutation tests to assess the statistical significance of the aggregated scores for the top genes discovered by the bivariate ranking. For each permutation, we randomly shuffled the data labels and recalculated the bivariate ranking. We carried out a total of 200 permutations, resulting in 100,000 (200 permutations multiplied by 500 genes per permutation) randomized aggregated scores.

### Selectivity

The selectivity is computed by taking the difference between the maximum and minimum CV scores obtained for each gene pair across the datasets. To enhance the sensitivity of this metric to score variations, we square each individual CV score before computing the difference. Specifically, for a given gene pair, let $S_1$, $S_2$, and $S_3$ represent the CV scores in the VR, MAYO, and GSE datasets, respectively. The selectivity metric $\sigma$ is then defined as:

1 $$\begin{aligned} \sigma =max(S_1^2,S_2^2,S_3^2)-min(S_1^2,S_2^2,S_3^2) \end{aligned}$$

## Supplementary Information

Supplementary Information.

## Data Availability

The VR dataset analysed during the current study is available in https://ars.els-cdn.com/content/image/1-s2.0-S0197458018303877-mmc2.xlsx^20^. The MAYO data that supports the findings of this study is available from the AD Knowledge Portal https://adknowledgeportal.synapse.org/, study *syn32141161*, but restrictions apply to the availability of these data, which were used under license for the current study, and so are not publicly available^21^. The GSE dataset analysed during the current study is available in Gene Expression Omnibus (GEO) Series GSE29378 https://www.ncbi.nlm.nih.gov/geo/query/acc.cgi?acc=GSE29378^2,61^.

## Code availability

The code used to run the analyses is available through Zenodo (10.5281/zenodo.13685511).
