# Supplementary material for: Cross study transcriptomic investigation of Alzheimer’s brain tissue discoveries and limitations
Source: Sci Rep. 2025 May 8;15:16041. doi: 10.1038/s41598-025-01017-y (PMC12062235; doi:10.1038/s41598-025-01017-y)
Supplement: Supplementary file 1 — Supplementary Information. [file 41598_2025_1017_MOESM1_ESM.pdf]

Cross study transcriptomic investigation of Alzheimer's brain tissue discoveries and limitations. Supplementary Information

Fernando Koiti Tsurukawa[1], Yixiang Mao[1], Cesar Sanchez-Villalobos[1], Nishtha Khanna[2], Chiquito J. Crasto[2], J. Josh Lawrence[3], and Ranadip Pal[1]

[1]Department of Electrical and Computer Engineering, Texas Tech University, Lubbock, 79409, TX, USA
[2]Center for Biotechnology and Genomics, Texas Tech University, Lubbock, 79409, TX, USA
[3]Department of Pharmacology and Neuroscience, Garrison Institute on Aging, Center of Excellence for Translational Neuroscience and Therapeutics, and Center of Excellence in Integrative Health, Texas Tech University Health Sciences Center, Lubbock, 79430, TX, USA
*Corresponding author: Ranadip Pal. Email: Ranadip.Pal@ttu.edu

Contents

1 Supplementary notes **3**
1.1 Differential expression 3
1.2 Feature Selection 3
1.3 L1 regularization 4

2 Supplementary tables **6**
2.1 Aggregated scores from top ranked genes 6
2.2 Relief comparison with differential expression analysis 7
2.3 Demographic information 8

3 Supplementary figures **9**
3.1 UMAP visualization 9
3.2 TSNE visualization 10
3.3 PCA visualization 11
3.4 Bar plot of mean cross-validation accuracy scores 12
3.5 Volcano plot of RNA-Seq datasets 13
3.6 Volcano plot of GSE dataset 14
3.7 KCNIP1 vs SLC38A2 15
3.8 RBP1 vs WNT7B 16
3.9 Permutation testing results (500 genes) 17

1 Supplementary notes

1.1 Differential expression

Out of the three hippocampal transcriptomic datasets, VR displays the most number of potential biomarkers using differential expression (DE) analysis. This is evidenced through by 1,538 genes being differentially expressed ($|\log_2 FC| \geq 1$) in VR, followed by MAYO dataset with 1,520 differentially expressed genes, 78 of which are differentially expressed in both VR and MAYO datasets. GSE, however, detected only 22 genes with significant fold change ratios between Alzheimer's Disease (AD) patients and control subjects. The significant difference is likely due to the type of technology used for measuring transcriptomic expression as GSE contains microarray data, whereas VR and Mayo contain RNA-Seq data. Studies have shown that RNA-Seq data can detect more differentially expressed transcripts as compared to microarray, especially genes with low expression.

Figures S6 and S7 present the volcano plots of the statistical results from the differential expression analysis for the VR, MAYO, and GSE datasets, alongside the top genes identified from the bivariate analysis. The plots highlight the significant differences in the shape of the scatter plots due to the p-value discrepancies between the studies. In the VR dataset, almost all genes exhibit exceptionally low p-values, indicating high statistical significance. In contrast, a significant percentage of genes in the MAYO dataset have high p-values, reflecting lower statistical significance. Despite these differences, both VR and MAYO datasets present a similar number of genes above the fold change threshold ($|\log_2| > 1$), which indicates a two-fold difference between the control and AD groups: 1,538 genes above this threshold for VR, and 1,520 for MAYO. The GSE dataset, derived from microarray data, shows a different magnitude of fold changes compared to VR and MAYO, highlighting the inherent variations in gene expression measurement between microarray and RNA-Seq technologies.

1.2 Feature Selection

Feature reduction techniques are regularly applied in transcriptomic data [3, 8]. Feature reduction can be achieved through feature extraction approaches such as Principal Component Analysis (PCA) [1], however, while PCA is excellent for reducing the dimensionality of data by capturing the maximum variance with a smaller set of orthogonal components, PCA provides limited interpretability of features in terms of genes or proteins. Other commonly used feature reduction approach is feature selection where a smaller set of features are involved in designing the predictive models. Some machine learning models have inbuilt feature selection (often grouped as embedded feature selection approach) such as Random Forests where the features that most reduce the cost function are selected in the node during the design of the classification or regression tree. In regression problems, linear models such as Lasso achieve feature selection through L1 regularization [9]. Explicit feature selection can also be achieved

through filter approaches such as correlation-based methods [2], differential expression [5] and Relief [4] that considers univariate relationships between features and the output response or through wrapper approaches such as sequential forward floating selection (SFFS) that considers the multivariate relationships among features and involves the ML model in feature selection [6, 7].

Relief is a nearest-neighbors based feature selection technique whose results significantly deviate from those differential expression analysis, we can observe this phenomenon further by applying Relief to select the top N ranking genes and check how many of them are significantly differentially expressed. (See Table S2)

Some of the top ranked genes from Relief present known biomarkers for AD, such as WNT7B, which participates in the neurodegeneration (*hsa05022*) and the pathway for AD (*hsa05010*) and acts in the Wnt signaling pathway (*hsa04310*), along with WIF1, another gene captured by our feature selection. Some genes selected from Relief participate in pre-established pathways in AD, indicating that Relief might be an alternative to differential expression analysis as a new univariate filtering approach.

Currently, several unique feature selection approaches have been proposed such as applying univariate filtering methods to rank each gene and to select gene subsets with good performance in terms of SVM classification. Univariate feature selection approaches are often used to initially narrow down the feature space followed by embedded or wrapper based feature selection to find multivariate alternatives to DE analysis.

1.3 L1 regularization

As an alternative to Relief based feature selection, we considered linear SVMs with L1 regularization to perform feature selection. L1 regularization techniques, such as Lasso (Least Absolute Shrinkage and Selection Operator), offer an effective approach for feature selection by imposing a constraint that encourages sparsity in the model coefficients. This helps identifying a subset of genes that are most relevant to the predictive models, reducing the dimensionality of the data. L1 regularization adds a penalty equal to the absolute value of the magnitude of coefficients to the loss function. The regularization parameter controls the trade-off between fitting the model well and keeping the coefficients small. As regularization parameter increases, more coefficients are shrunk to zero, resulting in a sparser model. By encouraging sparsity in the model coefficients, these techniques help in identifying the most relevant genes, thus reducing the dimensionality of the data and potentially improving the interpretability and performance of predictive models. The regularization parameter C was set to 0.10.

We observed that all genes selected through the linear SVM with L1 regularization were already present in the subset of genes previously selected using the Relief algorithm. This overlap indicates redundancy between the two feature selection methods. The coefficients obtained through L1 regularization in a linear SVM model are not guaranteed to have any causal interpretation. The

selected genes, while potentially useful for predictive modeling, should not be assumed to have direct causal relationships with the outcomes under study.

2 Supplementary tables

2.1 Aggregated scores from top ranked genes

Gene	Aggregated score	VR log2FC	MAYO log2FC	GSE log2FC
KCNIP1	322.23	-1.14	-0.51	-0.879
CA10	206.29	-1.22	-0.14	-1.06
CSPG5	174.48	-1.04	-0.28	-0.97
BCL6	157.95	0.92	0.48	0.463
SCG3	116.80	-1.25	-0.27	-0.756
CLK4	96.44	1.11	0.20	0.346
CXCL14	91.27	-1.17	-0.32	-1.01
STARD7	83.96	0.57	0.20	0.405
WNT7B	80.73	-1.14	-0.37	-0.595
SLC38A2	68.16	1.58	0.78	0.287

Table S1: **Aggregated scores and fold changes of top-ranked genes from the bivariate ranking methodology**. This table displays the aggregated scores of the top-ranked genes identified using our bivariate ranking methodology, alongside their respective fold changes in each of the three hippocampal studies (VR, MAYO, and GSE). The aggregated score is calculated by summing the cross-validation (CV) scores from training Support Vector Machines (SVMs) across all three datasets. Only gene pairs with CV scores above the 0.70 threshold were included to ensure high-performing pairs were considered. The top-ranked gene, KCNIP1, demonstrates a significant aggregated score, indicating its strong predictive power across the datasets.

2.2 Relief comparison with differential expression analysis

	Top N genes from Relief	Number of DE genes	Percentile
VR	10	6	60%
	50	21	42%
	100	38	38%
	200	59	30%
	300	76	25%
	400	94	23%
	500	111	22%
	1000	205	20%
MAYO	10	2	20%
	50	13	26%
	100	19	19%
	200	30	15%
	300	44	15%
	400	49	12%
	500	56	11%
	1000	94	10%
GSE	10	1	10%
	50	3	6%
	100	4	4%
	200	7	4%
	300	10	3%
	400	10	3%
	500	10	2%
	1000	12	1%

Table S2: **Comparison of significantly expressed genes among top ranked genes by Relief**. This table presents the number of significantly expressed genes identified among the top N ranked genes using the Relief algorithm, showcasing the distinction between our feature selection approach and traditional methods. Relief and its variants identify features by considering the importance of each gene in the context of its nearest neighbors, thereby capturing complex interactions and dependencies that might be overlooked by conventional methods. This nearest neighbor-based approach contrasts with the traditional univariate differential expression analysis, which evaluates each gene independently of others.

2.3 Demographic information

	Characteristic	Control	AD
VR	Samples	10	18
	Gender (% male)	5/10 (50%)	8/18 (44%)
	Age at death, yr. (±SD)	76 ± 12	75 ± 7
MAYO	Samples	15	20
	Gender (% male)	7/15 (47%)	7/20 (35%)
	Age at death, yr.	84 (80, 90)	81 (78, 88)
GSE	Samples	16	17
	Gender (% male)	11/16 (69%)	9/17 (53%)
	Age at death, yr. (±SD)	81.7 ± 6.9	77.3 ± 9.1

Table S3: **RNA-Seq cohort demographic information**. Data are presented as: sample size, gender (percentage of males) and age at death. Acronyms: VR=first study, MAYO=seconds study, GSE = third study, AD=Alzheimer's disease, yr=years, SD=standard deviation. Age values are mean ± standard deviation for VR and GSE and median (25th percentile, 75th percentile) for MAYO.

3 Supplementary figures

3.1 UMAP visualization

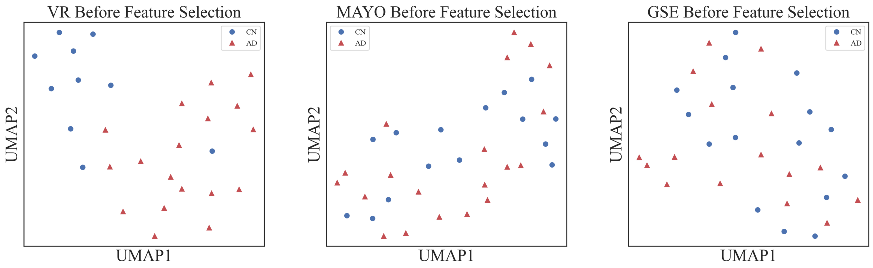

(a) VR (b) MAYO (c) GSE

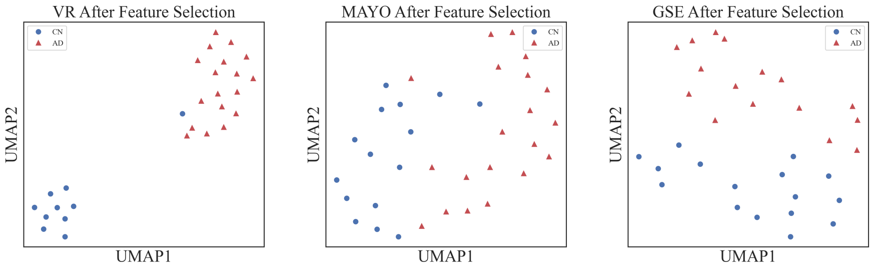

(d) VR (e) MAYO (f) GSE

Fig. S1: **Uniform Manifold Approximation and Projection (UMAP) visualizations from the three hippocampal datasets before and after feature selection** This figure presents the UMAP plots derived from the three distinct hippocampal datasets (VR, MAYO, and GSE) prior to the application of any feature reduction techniques and after feature selection. Each plot provides a two-dimensional representation of the high-dimensional gene expression data, illustrating the underlying clustering and separation patterns in each dataset.

3.2 TSNE visualization

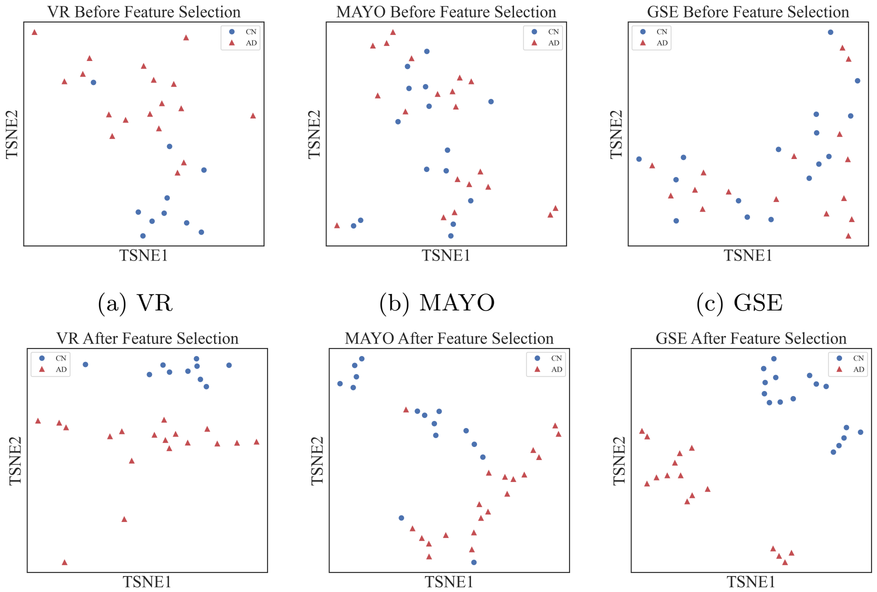

(a) VR (b) MAYO (c) GSE

(d) VR (e) MAYO (f) GSE

Fig. S2: **T-distributed Stochastic Neighbor Embedding (TSNE) visualizations from the three hippocampal datasets before and after feature selection**. The figure displays the reduced embedded space before and after the feature selection.

3.3 PCA visualization

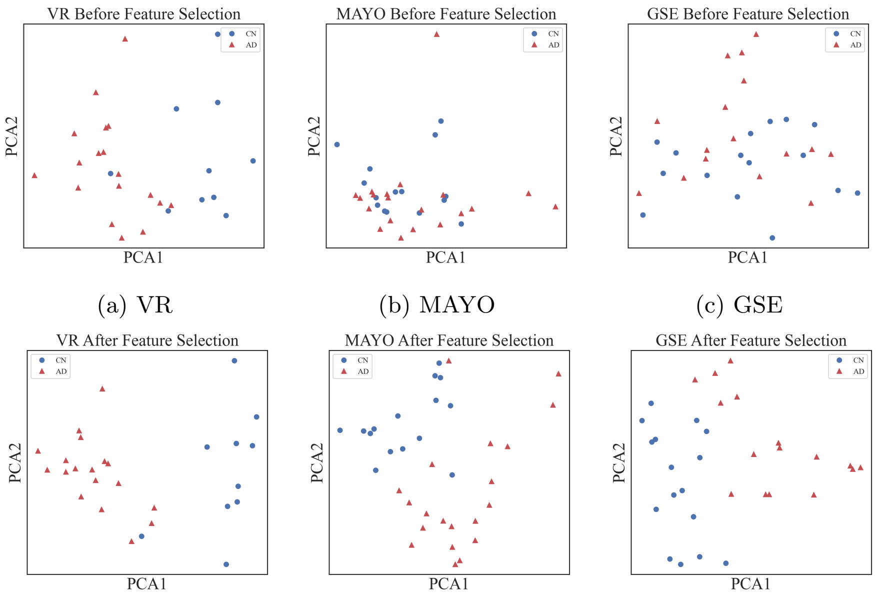

(a) VR (b) MAYO (c) GSE

(d) VR (e) MAYO (f) GSE

Fig. S3: **Principal Component Analysis (PCA) visualizations from the three hippocampal datasets before and after feature selection**. The figure displays the visual representation of each dataset using only the first two principal components from each dataset, before and after the feature selection.

3.4 Bar plot of mean cross-validation accuracy scores

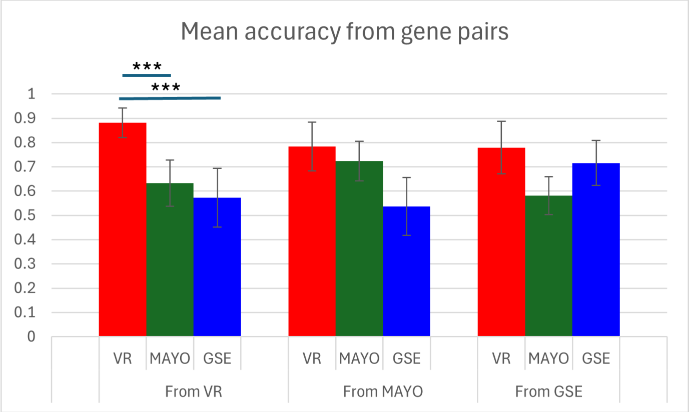

Fig. S4: **Bar plot of mean cross-validation accuracy scores for all gene combinations**. This figure displays a bar plot of the mean cross-validation accuracy scores for all possible combinations of the top 500 ranked genes in our bivariate ranking. The bars are color-coded to represent the accuracy scores in the three hippocampal datasets: red for VR, green for MAYO, and blue for GSE. "From VR," "From MAYO," and "From GSE" indicate that the gene pairs were selected based on feature selection applied within the respective datasets. Statistical significance of Mayo and GSE having lower mean accuracy as compared to VR was measured by Welch's t-test ($p < 0.001$)

3.5 Volcano plot of RNA-Seq datasets

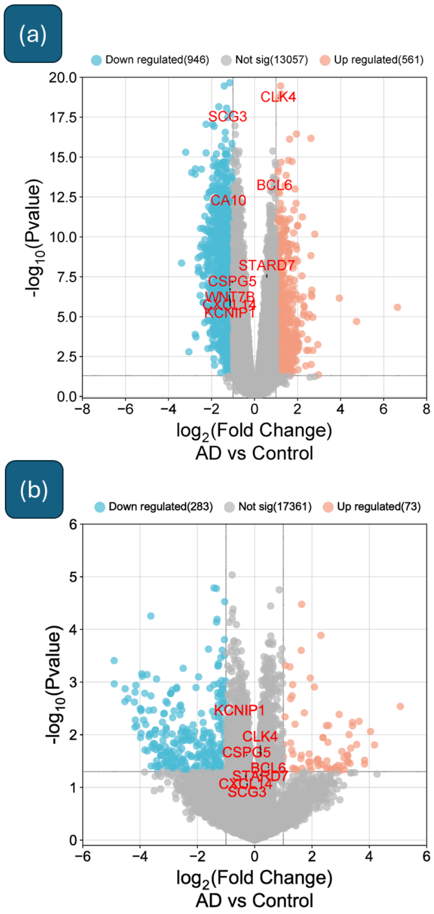

Fig. S5: **Differential expression analysis volcano plots for VR and MAYO datasets**. This figure presents volcano plots for the differential expression analysis in the VR and MAYO datasets. The x-axis represents the logarithm of the fold change for each gene, while the y-axis represents the negative logarithm of the p-value, indicating the statistical significance of the differential expression. The top genes identified by the bivariate ranking methodology are labeled in red.(a) The first plot illustrates the differential expression results for the VR dataset. Almost all genes in the VR dataset exhibit exceptionally low p-values (high negative logarithm). 1,538 genes exceed the fold change threshold ($|\log_2| > 1$), indicating a two-fold difference between the control and Alzheimer's Disease (AD) groups. (b) The second plot shows the differential expression results for the MAYO dataset. In contrast to the VR dataset, a significant percentage of genes in the MAYO dataset have high p-values, reflecting lower statistical significance. Nonetheless, 1,520 genes surpass the fold change threshold ($|\log_2| > 1$), with 78 DE genes in common with VR.

3.6 Volcano plot of GSE dataset

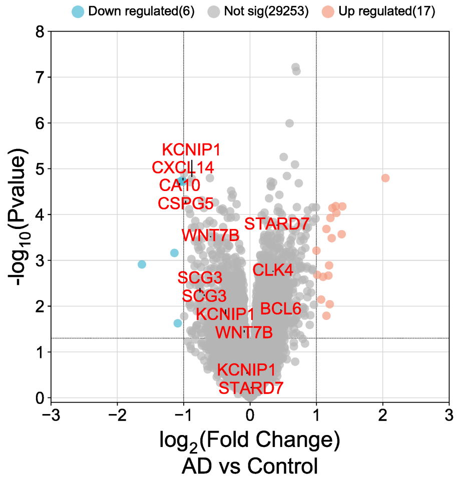

Fig. S6: **Volcano plot of differential expression analysis in GSE dataset**. This figure presents a volcano plot for the differential expression analysis in the GSE dataset, which is derived from microarray data. The x-axis represents the logarithm of the fold change for each gene, while the y-axis represents the negative logarithm of the p-value, indicating the statistical significance of the differential expression. The GSE dataset shows a distinctive distribution pattern, exhibiting a narrow range of fold changes, illustrating the inferior sensitivity of microarray technique.

3.7 KCNIP1 vs SLC38A2

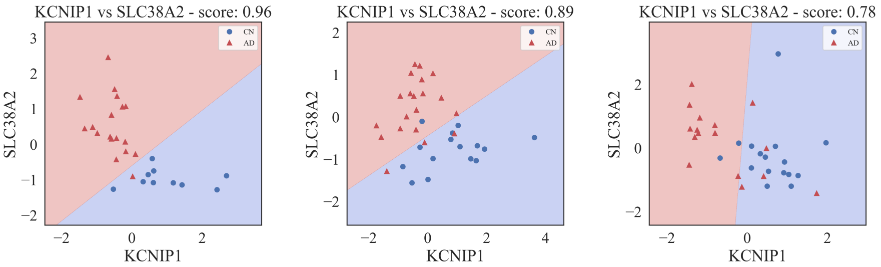

(a) VR (b) MAYO (c) GSE

Fig. S7: **Plots of gene pairs selected from bivariate ranking methodology**. This figure presents the performance of the gene pair KCNIP1 and SLC38A2 in differentiating Alzheimer's disease (AD) patients from control subjects across three hippocampal datasets (VR, MAYO, and GSE). The gene expression counts are used to train linear Support Vector Machine (SVM) classifiers. The first plot shows the SVM classifier's decision boundary for the VR dataset. The linear classifier effectively distinguishes between AD and control patients, with a clear decision boundary slope indicating the contribution of KCNIP1 and SLC38A2 expression levels in classification. The second plot shows the the decision boundary of the same gene pair in the MAYO dataset, mirroring the slope observed in the VR dataset. The third plot demonstrates the classifier's application to the GSE dataset. While the separation between AD and control patients is evident, the decision boundary's slope differs from those in the VR and MAYO datasets, highlighting dataset-specific variations in gene expression patterns.

3.8 RBP1 vs WNT7B

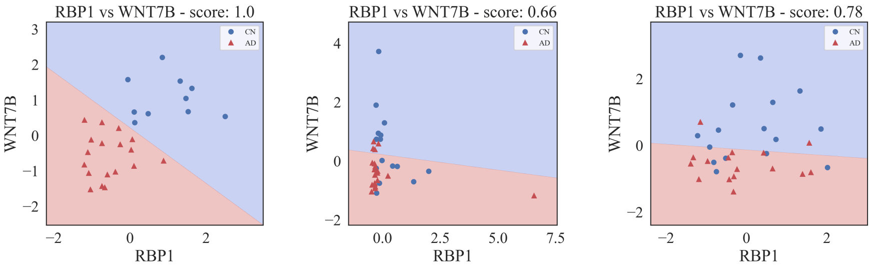

(a) VR (b) MAYO (c) GSE

Fig. S8: **Performance of genes RBP1 and WNT7B across different datasets**. This figure presents the plots of gene pairs RBP1 and WNT7B, highlighting their variable performance across the VR, MAYO, and GSE hippocampal datasets. The first plot shows the performance of RBP1 and WNT7B in the VR dataset. The gene pair performs well, effectively differentiating between AD and control patients, indicating strong predictive power in this dataset. The second plot depicts the performance in the MAYO dataset. Here, the gene pair fails to provide meaningful separation between AD and control patients. Interestingly, RBP1 appears upregulated in the MAYO dataset due to an outlier causing the AD distribution of RBP1 reads to be positively biased. The third plot illustrates the results for the GSE dataset. In this case, there is no improvement over univariate analysis, as WNT7B alone is already capable of splitting the data with similar accuracy, rendering the addition of RBP1 redundant.

3.9 Permutation testing results (500 genes)

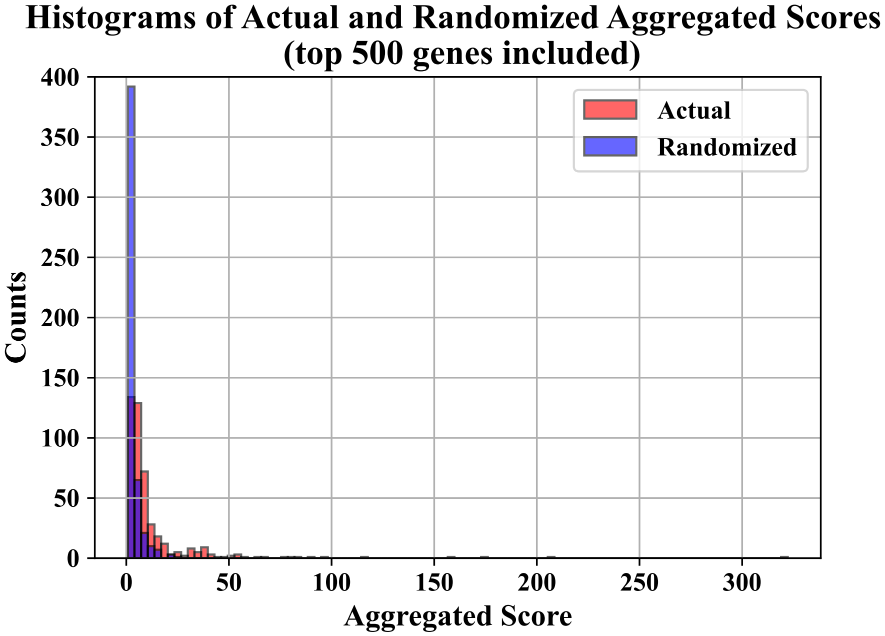

Fig. S9: **Histograms of the top 500 actual (red) and randomized (blue) aggregated scores**. The top 500 randomized aggregated scores were picked from 100,000 randomized aggregated scores generated by 200 permutations.

References

[1] Elena Galea, Laura D. Weinstock, Raquel Larramona-Arcas, Alyssa F. Pybus, Lydia Giménez-Llort, Carole Escartin, and Levi B. Wood. Multi-transcriptomic analysis points to early organelle dysfunction in human astrocytes in Alzheimer's disease. *Neurobiology of Disease*, 166:105655, 5 2022.

[2] Mark A Hall. *Correlation-based feature selection for machine learning*. PhD thesis, The University of Waikato, 1999.

[3] Zena M. Hira and Donald Gillies. A review of feature selection and feature extraction methods applied on microarray data. *Advances in Bioinformatics*, 2015:1–13, 6 2015.

[4] Igor Kononenko. Estimating attributes: Analysis and extensions of relief. In Francesco Bergadano and Luc De Raedt, editors, *Machine Learning: ECML-94*, pages 171–182, Berlin, Heidelberg, 1994. Springer Berlin Heidelberg.

[5] Jeffrey T. Leek, Eva Monsen, Alan R. Dabney, and John D. Storey. EDGE: extraction and analysis of differential gene expression. *Bioinformatics*, 22(4):507–508, 12 2005.

[6] Ranadip Pal. *Predictive modeling of drug sensitivity.* Academic Press, 11 2016.

[7] P. Pudil, J. Novovičová, and J. Kittler. Floating search methods in feature selection. *Pattern recognition letters*, 15(11):1119–1125, 11 1994.

[8] Yvan Saeys, Iñaki Inza, and Pedro Larrañaga. A review of feature selection techniques in bioinformatics. *Bioinformatics*, 23(19):2507–2517, 8 2007.

[9] Robert Tibshirani. Regression shrinkage and selection via the lasso. *Journal of the Royal Statistical Society. Series B (Methodological)*, 58(1):267–288, 1996.
